# Addressing sexual health after cancer: whose job is it anyway? Provider practices and communication barriers

**DOI:** 10.1007/s00520-026-11186-9

**Published:** 2026-09-22

**Authors:** Abby Girard, Eloise von DeWitt, G. Nic Rider, Melissa A. Geller, Rebekah Pratt, Rachel I. Vogel, Jennifer B. Reese

**Affiliations:** 1 Department of Family Medicine and Community Health, University of Minnesota, Minnesota, MN USA; 2 Division of Gynecologic Oncology, University of Minnesota, Minneapolis, MN USA; 3 Cancer Prevention and Control Program, Fox Chase Cancer Center, Philadelphia, PA USA; 4 School of Health in Social Sciences, University of Edinburgh, Edinburgh, UK

**Keywords:** Cancer survivorship, Sexual health, Provider communication, Quality of life

## Abstract

**Purpose:**

Despite established clinical guidelines recommending sexual health screening in oncology care, significant gaps in provider-patient communication persist. This study examined the experiences of gynecologic oncology providers regarding sexual health communication to guide the design of a targeted training intervention.

**Methods:**

Semi-structured interviews were conducted with 21 gynecologic oncology healthcare providers, including gynecologic oncologists, nurse practitioners, and physician assistants, practicing across three large healthcare systems in a single Midwestern U.S. state. Interviews were conducted via Zoom, audio recorded, professionally transcribed, and analyzed using inductive thematic analysis.

**Results:**

Two overarching themes were identified: contextual variability of sexual health communication and factors influencing sexual health communication. Providers described variability in the initiation, timing, and content of sexual health discussions, with advanced practice providers reporting greater comfort initiating these conversations than physicians. Key barriers included perceived time constraints, provider and patient discomfort, age-related assumptions, cultural and linguistic challenges, and uncertainty about professional scope of practice. Notably, providers demonstrated awareness of these barriers yet reported difficulty translating that awareness into consistent practice.

**Conclusion:**

Gynecologic oncology providers recognize the importance of sexual health communication but face substantial structural, interpersonal, and cultural barriers that limit consistent implementation. Findings support the need for accessible, skills-based training and system-level strategies that embed sexual health communication as a routine component of gynecologic cancer survivorship care.

## Introduction

During treatment and survivorship, gynecologic cancer survivors report rates of sexual dysfunction between 60% and 100% [1, 2]. Aerts and colleagues [3] noted that 83% of their sample of gynecologic cancer patients treated with surgery reported some kind of sexual dysfunction, with about half of survivors reporting two or more sexual dysfunctions. Gynecologic cancer survivors also grapple with shifts in self-perception, such as body image concerns, feeling unattractive, or perceived loss of femininity, all of which can affect their sexuality [4].

Since 2013, the National Comprehensive Cancer Network guidelines have included sexual health screening for people with cancer [5]. Additionally, in 2018, the American Society of Clinical Oncology published clinical practice guidelines for addressing sexual problems in people with cancer [6, 7]. Unfortunately, despite having established clinical care guidelines and care protocols [8], patients continue to report inadequate sexual health communication across their treatment trajectory [4, 9] and significant gaps in provider-patient sexual health communication. Effective communication between oncology providers and patients is positively associated with patient satisfaction, treatment adherence, and quality of life; yet, oncologists rarely receive training on effective communication [10–12]. Several studies related to cancer care have shown that effective patient-provider communication improves patients’ psychological adjustment to cancer and health outcomes [13–15] and that well-designed training programs can improve provider communication about sexual health and patient care experiences [14, 16].

Although addressing and prioritizing sexual health needs of patients can reduce the distress of sexual side effects caused by treatment [17, 18], physicians report lack of time, training, effective treatment options, personal discomfort, and perceived patient embarrassment as barriers [19–21]. In a study of gynecologic oncology providers, 98% of participants reported believing that sexual issues should be discussed, but only 21% raised the topic of sexual health with patients [22]. Studies have repeatedly indicated the importance of sexuality to a woman’s quality of life across the lifespan, and there is a growing recognition of the need for oncology providers to consistently and thoroughly integrate discussions about sexual and relational intimacy implications into patient care [23].

Training programs have been developed to improve oncologists’ communication, from intensive in-person training to brief mLearning interventions [24, 25]. While these programs have been found to improve communication in attendees, barriers around time, expense, and location often limit access and diminish adherence [12, 13]. While provider-focused interventions exist broadly, there continues to be a need within gynecologic cancer care to prioritize provider and patient sexual health education and communication to address significant sexual, relational and emotional health needs among survivors. The aim of this study was to explore the experiences of gynecologic cancer healthcare providers related to current practices of, barriers to, and facilitators of sexual health communication, with the ultimate objective of using the insights captured in this study to develop an intervention to train providers in sexual health communication.

## Methods

### Study design and setting

This qualitative study used semi-structured interviews and inductive thematic analysis to explore healthcare provider experiences with sexual health communication in gynecologic oncology practice. Reporting of the study was guided by the Consolidated Criteria for Reporting Qualitative Research (COREQ) 32-item checklist [26]. Participants were healthcare providers working primarily in gynecologic oncology clinics treating patients diagnosed with cervical, ovarian, endometrial, vulvar, or vaginal cancers. All participants practiced within three large healthcare systems located in a single Midwestern U.S. state.

### Participants and recruitment

Recruitment involved outreach from the study team to eligible participants via flyers and email invitations distributed within participating institutions. Interested providers accessed a study link which described the purpose of the study and informed them that interviews would explore their current sexual health communication practices and preferences related to sexual health communication and training. Interested providers accessed a study link that Interested providers completed a self-report eligibility and demographic survey via Qualtrics prior to interview participation. Participants meeting eligibility criteria were subsequently contacted by the study team to schedule their interview.

In total, 25 providers scheduled interviews and 21 (84%) completed study interviews. Participants who missed a scheduled interview were contacted by telephone and email on up to three occasions and offered the opportunity to reschedule. Those who did not respond following three contact attempts were classified as inactive. Participants who completed interviews received a $150 gift card in recognition of their time and contributions (Table 1).

**Table 1:** Participant demographics (*N* = 21)

Characteristic	*n* (%)
Age (years)	30–56 Mean age 44.7
Years in practice	1–25 Median 10 years
Gender identity	
Cisgender female	19 (90.5%)
Cisgender male	2 (9.5%)
Provider type	
Gynecologic oncologist	11 (52.4%)
Nurse practitioner	7 (33.3%)
Physician assistant	3 (14.3%)
Race/ethnicity	
Asian/Asian American	2 (9.5%)
Black/African American	1 (4.8%)
White	18 (85.7%)

### Data collection

Semi-structured interviews were conducted individually via Zoom by the principal investigator and research coordinator (A.G. and E.V.D.) and were audio and video recorded. No individuals other than the participant and interviewer were present during interviews Interviews lasted approximately 30 min on average, ranging from 25 to 40 min. The interview guide focused on current sexual health communication practices, provider perceptions of sexual health treatment, and preferences for educational or training resources. Formal field notes were not collected. Example interview questions are provided in Table 2.

**Table 2:** Example qualitative interview questions

Who usually brings these issues up? How is it done? [Probe: At what point in your patients’ treatment is it important to discuss sexuality with them]
Which kinds of sexual issues do you find that you are most likely to discuss with patients when it comes to sexuality? What about less likely? [Probe: Vaginal dryness? Libido? Pain or discomfort during sex? Poor body image? Intimacy concerns?] [Probe: Are there certain types of concerns you feel to be inappropriate to raise with patients? Why?]
When these issues come up (regardless of who initiates the conversation), how do you usually respond (what recommendations or resources do you provide)?
How do patient characteristics like age, sexual/gender orientation, culture, etc. …influence your comfort with discussing these issues with patients?
What gets in the way of or makes it hard for you to raise or address sexuality-related issues with your patients?

Data collection occurred between May and December 2024. A.G. and E.V.D. met weekly during data collection to discuss emerging concepts and progression of data collection. Recruitment and interviewing ceased when investigators (A.G and E.V.D.) determined that interviews were no longer generating new themes. No repeat interviews were conducted.

### Qualitative data analysis

Interviews were professionally transcribed verbatim, and transcripts reviewed against audio recordings by a research team member to ensure transcription accuracy. Corrected transcripts were uploaded into NVivo 10 software (QSR International) for analysis. Two research team members (A.G. and E.V.D.) conducted inductive thematic analysis to identify themes and subthemes across interviews [27]. An initial codebook was developed, and the first transcript was independently coded by both team members. Coding discrepancies were discussed and reconciled to achieve consensus and refine the coding framework. Subsequent transcripts were coded by the primary coder (A.G.) and cross-coded by a second coder (E.V.D.) to maintain consistency. Codes were subsequently organized into broader thematic categories based on conceptual similarity. Research team members met iteratively to finalize themes, subthemes, and representative quotations.

### Reflexivity and rigor

Interviews and primary analysis were conducted by A.G. and E.V.D. A.G. is a White cisgender woman, associate professor, licensed marriage and family therapist, and certified sex therapist with 13 years of clinical experience and research expertise in sexual health and oncology. E.V.D. is a White transgender/gender-diverse research assistant, licensed alcohol and drug counselor, and licensed professional clinical counselor with prior experience conducting sexual health research. Their clinical and research backgrounds provided familiarity with sensitive sexual health topics while also informing the perspectives they brought to data collection and interpretation. In particular, A.G.’s clinical and research expertise in sexual health represented an important positional influence, including an existing orientation toward sexual health as an important component of comprehensive cancer care.

Because of A.G.’s professional role at the University of Minnesota, some participants may have had prior professional interactions or working relationships with her. E.V.D. had no prior relationships with participants. A.G. and E.V.D. met regularly throughout data collection and analysis to discuss emerging findings, coding decisions, and alternative interpretations of the data. Coding discrepancies were discussed and resolved through consensus and reference to the source data (If accurate: An audit trail documenting code development and thematic decisions was maintained throughout analysis.)

Following primary analysis, G.N.R., a mixed-methods researcher whose research includes sexual and gender health and disparities in cancer care, reviewed the resulting themes and interpretations and provided an additional methodological and substantive perspective on the analytic framework. Other members of the author team did not participate in coding or analytic meetings.

### Ethical considerations

The study protocol and procedures were reviewed by the University of Minnesota Institutional Review Board and determined to be not human subjects’ research. Potential participants were provided with information about the study and given an opportunity to ask questions before agreeing to participate; written informed consent was not obtained. Participant information was de-identified during transcription and securely stored.

## Results

### Participants

Of the 21 gynecologic oncology healthcare providers (ages, 30–56 years, mean age of 44.7), 11 were gynecologic oncologists, and 10 were advanced practice providers (APPs) (Table 1). The majority of the sample self-reported identifying as female (90.5%). Their years of experience ranged from 1 to 25 years, with a mean of 10 years. Most (18; 85.7%) self-reported their racial identity as White, two (9.5%) self-reported being Asian/Asian American, and one (4.8%) self-reported being Black/African American.

### Interview findings

Two main themes were identified regarding gynecologic healthcare providers’ experiences with sexual health communication: (1) contextual variability of sexual health communication and (2) factors influencing sexual health communication. Within each of these overarching themes, multiple subthemes were identified. Each theme and subtheme is outlined below, along with illustrative quotes.

#### Theme 1: Contextual variability of sexual health communication

Gynecological healthcare providers commented on several aspects of their current clinical practices related to sexual health conversations and treatment recommendations. Three subthemes relating to provider practices were identified, including initiation and integration of sexual health communication in clinical practice, type of sexual health concerns discussed, and timing of sexual health communication.

##### Initiation and integration of sexual health communication in clinical practice

Participants described variability in how sexual health communication was initiated and integrated into clinical encounters, with practices shaped largely by provider role, scope of practice, and clinical timing. Responses varied based on expectations associated with provider roles. Gynecologic oncologists more often reported that sexual health communication was being addressed by advanced practice providers (APPs), particularly during postoperative and surveillance visits, whereas APP participants more frequently described initiating these conversations themselves. Professional role appeared to influence how sexual health communication was incorporated into care, wherein APPs more frequently described directly initiating sexual health discussions, particularly during postoperative, surveillance, and survivorship encounters, whereas gynecologic oncologists more commonly described these conversations in relation to treatment counseling or identified APPs as assuming a greater role in ongoing sexual health assessment.

One gynecologic oncologist explained, “So I would say in my practice, it’s really my [APPs] that do way more of that because they see them post-op and for surveillance” (Participant 11). In contrast, an APP participant reported, “I would say 80 percent of the time, it’s me… 20 percent of the time, it’s the patient.” (Participant 19).

Providers also described selectively initiating sexual health communication depending upon the patient populations. In this sample, providers reported being more likely to provide counseling and education about sexual side effects to younger patients, particularly those who were pre- or perimenopausal prior to treatment, as well as patients who underwent treatments resulting in significant anatomical changes. One participant noted providing counseling more commonly with those who, “were premenopausal before they had cancer” (Participant 12). Participants further described how sexual health communication emerged within routine clinical workflows. For providers whose work was primarily surgical, conversations were often limited to postoperative expectations. For others, sexual health communication was incorporated into routine examinations or survivorship visits, often prompted by clinical findings or symptom review. For example, one provider described initiating discussion during pelvic exams when physical findings suggested potential sexual concerns:

And especially if you’re doing the exam and sometimes the patient hasn’t brought it up at all, but I’ll be doing the exam and I’ll notice they have really severe vaginal atrophy. And I’ll say, now, are you having any pain or dryness that you’ve noticed? And then I can say, here’s what I’m seeing in your exam. Do you experience that as well? And a lot of times, that opens it up. (Participant 13)

Providers who continued to see patients following completion of active treatment often described sexual health communication as part of routine survivorship check-ins, with both providers and patients raising concerns. As one participant explained:

…So I’m asking them if there are any sexual health concerns, especially I would say more so after they’ve completed their treatments…patients will bring up their concerns or if they’re on endocrine therapies then they may bring it up. (Participant 2)

Across interviews, sexual health communication initiation therefore appeared to depend not only on provider comfort or initiative, but also on clinical context, visit purpose, and assumptions about which patients were most likely to benefit from these discussions.

##### Types of sexual health concerns discussed

Participants were asked to reflect on the types of sexual issues that come up in practice. All participants indicated patients raised topics of sexual dysfunction, primarily dyspareunia (pain during sex), loss of lubrication/arousal, and changes to sexual desire. For instance, one participant described,

So, there’s pain with sex. So, there’s dyspareunia. And then there’s lack of libido. And sometimes if it’s been, for instance, like a vulvar cancer and they’ve had a vulvectomy, sometimes there’s inability to achieve an orgasm. (Participant 20)

Additional topics that were commonly reported related to alterations in sexual function due to surgeries such as vulvectomy or changes after pelvic radiation. Several participants commented on differences in their comfort discussing issues they deemed physical, such as pain, versus conversations that related to body image, attraction, and desire:

Well, secretly, I always hope that it’s a vaginal pain issue because I have more tools in my toolbox that I can help actually make that better, whereas …when you kind of tease out the whole thing, it’s not that they don’t want to have sex, it’s that they don’t want to have sex with him. (Participant 4)

##### Timing of sexual health communication

Similar to the question of how sexual health communication is presented, the question of *timing* was explored in the interviews. Similar to the variability in initiation of sexual health communication, when these topics are introduced was also dependent on the provider’s role (gynecologic oncologist, or APP). For one MD, they reflected,

I’d say the majority of the sexual health concerns that we see are usually once the patients are in the surveillance portion of their treatment. It doesn’t come up a whole lot during treatment because they’re so focused on getting well and treating the disease. But when I see patients for surveillance visits, I’d say it comes up a large majority of the time. (Participant 1)

For many providers in this sample, there was a general consensus that sexual health communication is helpful at all stages, but is often reserved for after active treatment, and is more typically addressed in surveillance and survivorship visits where the focus is on symptom management and quality of life. One participant explained:

... It tends to come up a little bit later, meaning patients have finished all the treatment, they’re done, they’re in a different mode. Maybe one term used is survivorship mode or we’re in monitoring mode, and it tends to come up there. I oftentimes will -- sometimes it comes up during the pelvic exam. (Participant 3)

#### Theme 2: Factors influencing sexual health communication

The second overarching theme focused on factors that influence provider-initiated sexual health communication. These included subthemes of patient age, cultural influences, patient or provider discomfort and taboos around sexuality, and perceptions of time.

##### Patient age may influence discussions

Participants were asked to identify factors that might contribute to them not engaging in sexual health communication, or biases that may inform how they address sexuality, more generally. For several participants, the age of the patient was a determining factor or implicit bias that was identified. These age-related assumptions and selective communication were described by providers across varying levels of clinical experience, including relatively early career clinicians, with no clear descriptive pattern suggesting that these assumptions were limited to providers with longer practice histories. Many participants indicated that they engage in sexual health communication more consistently with younger patients:

I would say age is probably a really big one where … -- I find myself more likely to raise it with younger patients, actually, whereas older patients, I see more likely to wait for them to bring it up. (Participant 4)

The majority of participants also noted that their patient population tends to be older (post-menopausal), and that whether they are conscious of this or not in the moment, this age-related stigma contributes to assumptions about the patients’ sexuality. This was illustrated by Participant 8’s comments:

I think there’s a pretty big percentage of patients that are elderly or even post-menopausal who are not sexually active either because I’ve asked them or because I make the assumption that they are elderly and single. So, right or wrong I make that assumption and we don’t ever really talk about it probably at any point.

##### How culture influences sexual health communication

Additional factors that contributed to a lack of sexual health communication centered around discomfort or uncertainty when working with specific populations outside of the provider’s own culture. This could be due to the need to use language interpreters or family members in clinic visits, or to having limited knowledge of various cultural practices and attitudes about sexual health communication, as described by participant:

Sometimes you will find kind of that cultural aspect of that, you ask the woman, my patient, the question, whatever it is, and the husband or the male figure in that culture will be answering all of the questions. And so that becomes a little bit trickier. And you kind of notice that that’s something that they don’t always want to talk about. Certainly, they’ve never, like I’m thinking the Somali population, they don’t bring it up. And so usually when I ask, and of course, oftentimes, it’s through an interpreter, which I’m assuming the interpreter is interpreting my question correctly, and I don’t know what they’re exactly saying, but usually the response is something, oh, no, it’s not a concern. (Participant 6)

Other participants discussed the cultural attitudes of their state/region, where there is an expectation not to complain or talk about personal matters, and to avoid discomfort, described by Participant 11:

I think having worked on the east coast for most of my training, it’s definitely a little bit in the Midwest, people are more hesitant to talk about sex, especially even with their doctors.

##### Discomfort/taboo with sexual health

Both provider and patient discomfort were a common theme that came up when discussing barriers to sexual health communication. This included one participant’s general hesitance to talk about sex with:

I don’t have any issue talking about any particular topic, but there’s always that discomfort when you don’t know how comfortable someone’s going to be talking about that, of being the one to open that conversation up. (Participant 16)

For other participants, this discomfort manifested as avoidance of sexual health topics based on the patient’s identity:

I think what we realize, even as a group, is that we have quite a few… lesbian couples, and in that time period …we realize we didn’t actually ask or address sexual function for them. (Participant 21)

For many, discomfort and taboo intersected with biases related to age, relationship status, and culture.

##### How time influences how sexual health communication is prioritized

For the gynecologic oncologist participants, the perception of limited time during clinic encounters was the most prevalent barrier to discussions about sexual health communication, illustrated here:

… I have 15 to 30 minutes at the most and we’re talking about prognosis and we’re talking about the drugs and the treatment and the genetic testing. … There’s so many things to go through that it’s not something that I routinely bring up. (Participant 9)

This was consistent across all three clinical settings, despite vastly different practices. Many medical oncologists referenced the difference in visit duration between MD and APP appointments, suggesting that the “right” place for sexual health communication is with APPs during surveillance and survivorship due to the extended length of these visits

We try to set them up with survivorship visits with our APPs and they tend to delve into that much more frequently than we do. Because they just have the time. They have like an hour visit to address all the survivorship issues. (Participant 3)

In combination with the well-documented perceived time constraints, sexual health communication was seen as a lower priority than many other cancer-related concerns. For example, for MDs who see patients every 15 min and need to cover many treatment-related symptoms, the perception is often that sexual health concerns come last. In other situations, participants indicated that they plan to bring it up, but the patients present with a list of other issues that take priority. In both situations, sexual health communication is overshadowed by medical issues perceived as being more time-sensitive, as seen here:

Probably the biggest barrier, too, is you’re just dealing with a lot of other things and appointments. And it kind of feels like maybe the bottom of the list or whatever of things, but it might not be the bottom of the list for the person. But, in your mind, you’re thinking about all of these other side effects or symptoms you should be talking about. (Participant 15)

##### How perceived scope of practice influences communication

The final factor that influenced sexual health communication is related to the concept of “who’s job is this?” For many providers, they felt like sexual health communication was an important topic in this population, but not necessarily something they should be addressing.

…I don’t feel like I have the actual authority nor the background to kind of take that next step to address sexual desire in that regard. (Participant 20)

Others felt competent to open the conversation but indicated that without the knowledge and resources to provide solutions, they were out of their scope, practically speaking, such as with Participant 1 below:

No, I think probably I’m more comfortable with physical difficulties than I am emotional and relational. For me, the physical is what I -- is what I do. I mean, that’s what I trained to do and that’s my background. The emotional I think can be much more difficult. And I’ll be honest, I don’t get into that. That’s a very -- that can be a very intense topic.

## Discussion

This study explored gynecologic oncology providers’ experiences and perceptions of sexual health communication within clinical care. Across disciplines, participants recognized sexual health as an important dimension of survivorship yet described substantial variability in when and how these discussions occurred. The findings reinforce persistent discrepancies between guidelines advocating for sexual health communication in oncology and the realities of limited implementation in practice. Critically, this study extends prior work by providing granular, role-differentiated accounts of *how* these gaps operate in practice, moving beyond documenting that gaps exist to illuminating the interpersonal, structural, and cultural mechanisms that sustain them.

### Provider-initiated and selective communication

Consistent with prior studies [28, 29], healthcare providers perceived that sexual health communication was generally provider-initiated, though the initiating clinician often depended on professional role. APPs seemed more likely to initiate sexual health discussions, particularly during post-treatment and survivorship visits, while physicians tended to report deferring these conversations to APPs or other team members. While role-based delegation in oncology teams has been previously described in general communication literature [11, 14], this study offers a more nuanced finding: delegation is not simply a structural convenience but is actively rationalized by physicians as appropriate scope-of-practice behavior in the context of other demands placed on physicians, a distinction that shifts the narrative from a resourcing problem to a perception problem with implications for how training interventions should be designed. Without reframing sexual health communication as a shared clinical responsibility, delegation risks creating systematic gaps in care for patients who may not have access to extended survivorship visits.

Providers were also more likely to discuss sexual health with younger or premenopausal patients and those who had undergone treatments that directly altered sexual anatomy or function. This selective counseling mirrors prior findings of age-related bias in oncology care, where older survivors and those without partners are less frequently engaged in discussions about sexuality [1, 30–32]. What this study adds is providers’ own candid articulation of the reasoning behind this bias, including explicit acknowledgment that they make assumptions about older or single patients’ sexual activity without asking. This self-reported reflexivity is notable: providers are not unaware of these patterns, yet awareness alone has not translated into changed behavior, suggesting that bias-focused training alone may be insufficient without addressing the additional barriers.

Age-related assumptions and selective communication were evident among providers across levels of clinical experience, including relatively early-career clinicians. Even though the present study was not aimed at determining the origins of these beliefs, this finding suggests that age-related assumptions about sexuality may not be attributable solely to generational differences among clinicians. Rather, they may reflect broader professional norms and gaps in education regarding sexual health and sexuality across the lifespan. Prior research has identified substantial gaps in sexual health education among medical trainees [33], as well as limited preparation among practicing clinicians to address sexuality in later life [34]. These educational gaps may leave clinicians inadequately prepared to recognize and address sexual health concerns across patient populations and across the lifespan. Accordingly, interventions should address not only clinicians’ communication skills, but also assumptions about age, sexual activity, and which patients are perceived as needing sexual health assessment.

### Barriers to sexual health communication

Participants identified multiple overlapping barriers to sexual health communication, including perceived time constraints, discomfort, cultural uncertainty, and questions of professional role. Lack of time emerged as the most significant limiting factor, particularly for physicians managing high patient volumes and complex treatment discussions. Similar to previous work [7, 14, 21, 29], participants reported that sexual health often fell to the bottom of the clinical agenda. Treatment decisions and symptom management are often prioritized, potentially minimizing the potential importance of sexuality for patients’ wellbeing and how closely tied sexuality can be to patients’ quality of life, adherence, and psychological adjustment. Although clinicians often discussed feeling time constraints as a limiting factor in their communication and as a reason for deprioritizing sexual health in their patient visits, research suggests that discussions of sexual health can in fact be effective while also being brief. This finding suggests that interventions for clinicians need to address time constraints and encourage models of communication that providers can accomplish in a brief amount of time [24, 25]. This framing has direct implications for intervention design: reducing time pressure alone may be insufficient if providers do not also reconceptualize sexual health as a non-optional component of survivorship care.

Discomfort and perceived taboo further constrained communication. Even providers who considered themselves comfortable discussing sexual health reported hesitancy due to uncertainty about patient comfort with the topic or cultural sensitivity. These findings are consistent with research demonstrating that mutual embarrassment and lack of shared language continue to impede open dialogue about sexuality in oncology [8, 22, 35, 36]. A meaningful addition from the current data is providers’ differentiation between comfort with physical concerns, such as pain or atrophy, versus emotional and relational concerns such as desire, intimacy, and body image. This distinction is not well-captured in prior work and suggests that existing comfort levels are content-specific, rather than global, with important consequences for training design: programs that focus exclusively on physical symptom management may leave providers underprepared for the relational dimensions of sexual health that patients frequently raise.

Cultural and linguistic barriers added another layer of complexity. Providers described challenges using interpreters, navigating family involvement, and addressing cultural norms that discourage open sexual discussion. These barriers echo earlier work emphasizing the need for culturally responsive training in sexual health communication [37, 38]. Extending this literature, the current study reveals that gynecologic cancer providers often have limited insight into whether their questions are being accurately interpreted or whether patients from certain cultural backgrounds are simply deferring rather than genuinely reporting no concerns, suggesting a potential blind spot that warrants specific attention in training curricula. Several participants also recognized heteronormative bias in their own practice, noting that same-sex couples were sometimes overlooked in discussions about sexual function, a gap that has received relatively little attention in gynecologic oncology-specific literature and that this study helps to surface.

### Implications for practice and training

Despite the inclusion of sexual health assessment in NCCN and ASCO survivorship guidelines [39, 40], translation into practice remains inconsistent. The current findings highlight the variability and inconsistency of screening practices, which suggests a need for pragmatic strategies that embed sexual health communication within routine workflows. Brief screening questions integrated into electronic medical records could normalize sexual health discussions and prompt providers to initiate dialogue during visits, although whether these would be sufficient to prompt useful discussions requires investigation.

Training initiatives should move beyond raising awareness to building skills, confidence, and providing resources. Notably, this study suggests that awareness of bias, discomfort, and time constraints already exists among many providers. At the same time, knowledge gaps still exist, as was evident when discussing comfort with more nuanced sexual health themes, such as sexual desire. Self-reflective modules addressing implicit bias, language use, and patient-centered communication could enhance clinician competence and comfort, but should be paired with concrete communication tools and practice scenarios that build behavioral confidence alongside content to increase knowledge of sexual issues and management strategies. Because time and cost remain key barriers to in-person training, virtual or asynchronous educational programs tailored to gynecologic oncology may be particularly beneficial [7, 10, 12]. Embedding sexual health education within tumor boards, case conferences, and continuing medical education opportunities could further reinforce its importance as a routine element of high-quality supportive care.

### Future directions and intervention

Future research should investigate how provider-reported practices align with patient experiences within the same clinical systems. Studies assessing both patient and provider perspectives would clarify whether gaps arise from provider avoidance, patient hesitancy, systemic constraints, or a combination thereof. Intervention trials testing the implementation of brief, structured sexual health communication protocols could evaluate effects on patient satisfaction, distress, and relational outcomes. Finally, expanding research to include racially, culturally, and geographically diverse samples is essential to ensure that sexual health communication strategies are equitable and responsive across populations.

### Limitations

This study was conducted with providers from three large healthcare systems within a single Midwestern U.S. state, which may limit generalizability. Participants were predominantly female and White, which limited our ability to examine how clinicians’ intersecting racial/ethnic and gender identities may shape sexual health communication. The small number of participants from minoritized racial/ethnic and gender groups precluded meaningful subgroup comparisons and, within this relatively small professional community. Future studies should purposively recruit more diverse provider samples to examine how intersecting provider identities and professional roles influence sexual health communication. In addition, those opting into interviews may have had greater interest or comfort with sexual health communication than those who did not opt-in to participating, although the candidness with which participants described substantial difficulties in raising sexual health with their patients lends credibility to the findings. Nonetheless, the consistency of identified barriers across roles and settings suggests that these challenges are broadly representative within gynecologic oncology.

## Conclusion

Sexual health communication remains a critical but under-addressed element of gynecologic cancer survivorship care. Our findings suggest that providers often recognize its importance yet encounter structural, interpersonal, and cultural barriers that restrict consistent practice. Addressing these gaps will require system-level changes that integrate sexual health communication into standard workflows, provide efficient and accessible training, and promote multidisciplinary collaboration. Sustained institutional commitment to sexual health as a component of supportive care is essential to improving quality of life for gynecologic cancer survivors.

## Data Availability

No datasets were generated or analyzed during the current study.

## Declarations

### Disclaimer

The funder had no role in study design, data collection, analysis, or decision to publish.
